# Evaluation of an assay for methylated *BCAT1* and *IKZF1* in plasma for detection of colorectal neoplasia

**DOI:** 10.1186/s12885-015-1674-2

**Published:** 2015-10-06

**Authors:** Susanne K. Pedersen, Erin L. Symonds, Rohan T. Baker, David H. Murray, Aidan McEvoy, Sascha C. Van Doorn, Marco W. Mundt, Stephen R. Cole, Geetha Gopalsamy, Dileep Mangira, Lawrence C. LaPointe, Evelien Dekker, Graeme P. Young

**Affiliations:** 1 Clinical Genomics Pty Ltd, Sydney, Australia; 2 Flinders Centre for Innovation in Cancer, Flinders University of South Australia, Adelaide, Australia; 3 Bowel Health Service, Repatriation General Hospital, Adelaide, Australia; 4 Academic Medical Centre, Amsterdam, The Netherlands; 5 Flevo Hospital, Almere, The Netherlands

**Keywords:** DNA methylation, Screening, Colorectal cancer, *BCAT1*, *IKZF1*

## Abstract

**Background:**

Specific genes, such as *BCAT1* and *IKZF1,* are methylated with high frequency in colorectal cancer (CRC) tissue compared to normal colon tissue specimens. Such DNA may leak into blood and be present as cell-free circulating DNA. We have evaluated the accuracy of a novel blood test for these two markers across the spectrum of benign and neoplastic conditions encountered in the colon and rectum.

**Methods:**

Circulating DNA was extracted from plasma obtained from volunteers scheduled for colonoscopy for any reason, or for colonic surgery, at Australian and Dutch hospitals. The extracted DNA was bisulphite converted and analysed by methylation specific real-time quantitative PCR (qPCR). A specimen was deemed positive if one or more qPCR replicates were positive for either methylated *BCAT1* or *IKZF1* DNA. Sensitivity and specificity for CRC were estimated as the primary outcome measures.

**Results:**

Plasma samples were collected from 2105 enrolled volunteers (mean age 62 years, 54 % male), including 26 additional samples taken after surgical removal of cancers. The two-marker blood test was run successfully on 2127 samples. The test identified 85 of 129 CRC cases (sensitivity of 66 %, 95 % CI: 57–74). For CRC stages I-IV, respective positivity rates were 38 % (95 % CI: 21–58), 69 % (95 % CI: 53–82), 73 % (95 % CI: 56–85) and 94 % (95 % CI: 70–100). A positive trend was observed between positivity rate and degree of invasiveness. The colonic location of cancer did not influence assay positivity rates. Gender, age, smoking and family history were not significant predictors of marker positivity. Twelve methylation-positive cancer cases with paired pre- and post-surgery plasma showed reduction in methylation signal after surgery, with complete disappearance of signal in 10 subjects. Sensitivity for advanced adenoma (*n* = 338) was 6 % (95 % CI: 4–9). Specificity was 94 % (95 % CI: 92–95) in all 838 non-neoplastic pathology cases and 95 % (95 % CI: 92–97) in those with no colonic pathology detected (*n* = 450).

**Conclusions:**

The sensitivity for cancer of this two-marker blood test justifies prospective evaluation in a true screening population relative to a proven screening test. Given the high rate of marker disappearance after cancer resection, this blood test might also be useful to monitor tumour recurrence.

**Trial registration:**

ACTRN12611000318987.

**Electronic supplementary material:**

The online version of this article (doi:10.1186/s12885-015-1674-2) contains supplementary material, which is available to authorized users.

## Background

Colorectal cancer (CRC) is the second leading cause of death from cancer in the developed world [1]. Randomised controlled trials (RCT) in the general population have shown that early detection by screening, such as with faecal occult blood test (FOBT) or flexible sigmoidoscopy, reduces mortality and may also reduce incidence [2–6]. Reduction in mortality is dependent on treatment of curable neoplasms destined to cause death while reduction in incidence is dependent on detection and removal of pre-invasive lesions (i.e. adenomas). Given that early detection of a neoplasm is worthwhile for either a bleeding phenotype or a phenotype that enables visualisation (as detected by FOBT and flexible sigmoidoscopy, respectively), detection of a neoplasm based on other factors such as molecular characteristics may have the same benefit, but this is yet to be established.

In addition to the ability of a test to detect early curable lesions, a screening test can only be effective if the targeted individual undertakes the test. This behavioural consideration presents certain barriers for endoscopic methods and in some countries also for FOBT. Participation rates for both FOBT and endoscopic methods are highly variable and clearly sub-optimal in many settings [7].

It has been suggested that a blood test would be more acceptable and circumvent some of the barriers with established screening methods [8, 9]. A blood test could be deployed as an alternative frontline screening test or else as a “rescue” strategy that aims to engage those who reject the existing RCT-proven methods such as FOBT and flexible sigmoidoscopy. The appropriate manner of deployment will depend in part on the accuracy of such a blood test.

Aberrant DNA methylation is a characteristic of colorectal tumours [10, 11]. *SEPT9* is one such tumour marker methylated in colorectal neoplasia that is detectable in blood [12, 13], but its clinical performance as a screening test is suboptimal. We have previously reported the identification and validation of a cohort of genes with hypermethylated regions that show promise for differentiating adenomas and early stage cancer from normal state and benign pathology [14]. More recently, we have shown that cell free circulating DNA extracted from blood from CRC patients has a significantly higher fraction of methylation across two genes, namely *BCAT1* and *IKZF1*, compared to normal controls [15]. It is important to determine the accuracy of detecting methylated *BCAT1* and *IKZF1* DNA in blood across the range of neoplastic lesions encountered in the colon before proceeding to compare outcomes from screening programs using the two-marker blood test, to programs using proved screening tests. The latter step is crucial to the inclusion of tests based on blood molecular markers in screening programs since early detection alone does not guarantee program efficacy or effectiveness when the biological basis of lesion detection is different [16, 17].

The goal of this study was to estimate true and false positive rates of the two-marker blood test for screen-relevant stages of colorectal neoplasia, namely advanced adenoma and CRC of specific stage, and across the full spectrum of non-neoplastic pathologies encountered in the colon/rectum when screening a large population.

## Methods

### Study overview

This was a multi-centre predominantly prospective study funded in part by the National Health and Medical Research Council (NHMRC) and Clinical Genomics Technologies Pty Ltd (CGT) to estimate the sensitivity and specificity of a test detecting methylated *BCAT1* and/or *IKZF1* DNA in blood from people with neoplasia or non-neoplastic pathologies likely to be encountered in the colon and rectum. Findings at colonoscopy were used as the diagnostic standard. The study was approved by the Southern Adelaide Clinical Human Research Ethics Committee (April 4, 2005) and Medical Ethical Board of Academic Medical Centre Amsterdam (July 12, 2011). Written informed consent was obtained from all recruits prior to any procedures. Clinical and research staff at the medical institutions audited clinical data and verified case classification blinded to assay results determined by CGT. The clinical data were only released subsequent to completion of testing of all collected samples. Test results were not disclosed to subjects or their physicians. The trial is registered at Australian and New Zealand Clinical Trials Registry trial registration number 12611000318987.

### Population

Subjects aged 33-85 years old and either scheduled for colonoscopy for standard clinical indications (prospective element), or shown at colonoscopy within the prior ten days to have CRC that had not been treated (retrospective element), were approached about volunteering for the study. The participating centres were Repatriation General Hospital (Daw Park, South Australia), Flinders Medical Centre (Bedford Park, South Australia), Academic Medical Centre (Amsterdam, The Netherlands) and Flevo Hospital (Almere, The Netherlands). Following enrolment, cases were excluded if the scheduled colonoscopy was cancelled or if insufficient blood was available.

### Clinical procedures

Venous blood was collected into two 9mL K3EDTA Vacuette tubes (Greiner Bio-One, Frickenhausen, Germany) from subjects either prior to them being sedated for colonoscopy but after consumption of bowel preparation solution, or prior to preparation for surgery but following colonoscopic diagnosis. A second sample was obtained from 26 CRC cases one month or more after surgery. Blood tubes were kept at 4 °C until commencing plasma processing. Plasma was prepared within 4 hours of blood collection by centrifugation at 1,500 g for 10 minutes at 4 °C (no braking), followed by retrieval of the plasma fraction and a repeat centrifugation. The resulting plasma was stored at -80 °C. Frozen plasma samples were shipped on dry ice to CGT and stored at -80 °C until testing.

No study-wide control of colonoscopy or pathology procedures or quality was undertaken as the study aimed to assess marker performance relative to outcomes determined in usual clinical practice. All procedures were performed by hospital-accredited specialists and so met site-specific standards for sedation, monitoring, imaging, and equipment. Histopathology and staging of neoplasia used routine procedures at each clinical site. Cases were excluded if any data crucial to clinical diagnosis was not obtainable, e.g. if colonoscopy was incomplete.

### Pathological classification

An independent physician assigned diagnosis for all cases used in this study on the basis of colonoscopy, surgical and histopathological findings. CRC was staged according to AJCC 7th Edition [18]. Advanced adenoma was defined as adenoma with any of the following characteristics: (a) ≥ 10 mm in size, (b) >20 % villous change, (c) high grade dysplasia, or (d) serrated pathology. Cases with more than two tubular adenomas or stage 0 cancer were also classified as advanced adenoma. Non-advanced adenoma refers to those not meeting the characteristics of an advanced adenoma. Hyperplastic polyps were classed as non-neoplastic pathologies. Where multiple pathologies were present, the most advanced neoplasm was used as the principal diagnosis. Location of the principal neoplasm was defined as that of the most advanced lesion in a patient with multiple neoplasms. Where multiple non-neoplastic diagnoses were present, the principal diagnosis was allocated in the following hierarchy (descending): inflammatory bowel disease (IBD), hyperplastic polyp, angiodysplasia, haemorrhoids, diverticular disease.

### Test method

All plasma samples of at least 3.9mL were assayed for the presence of methylated *BCAT1* and *IKZF1* DNA at CGT’s laboratories by trained and qualified staff blinded to clinical results (see Additional file 1 for details). Samples were analysed in batches of 22 clinical samples and two process controls. Batches were loaded on a QIASymphony SP instrument (Qiagen, Hilden, Germany) and cell-free DNA was extracted using a QIASymphony Circulating Nucleic Acid Kit (Qiagen, Hilden, Germany) according to manufacturer’s instructions (Additional file 1). The extracted DNA was bisulphite-converted using the EpiTect Fast Bisulfite Conversion kit (Qiagen) and QIACube instrument (Qiagen) as recommended by manufacturer but with minor modifications (see Additional file 1). The resulting bisulphite-converted DNA was analysed as three replicates in a triplex real-time qPCR assay (*ACTB* control, methylated *BCAT1* and *IKZF1*) performed on a Roche LightCycler 480 Model II instrument (see Additional file 1). A sample was deemed positive if at least one qPCR replicate was positive for either *BCAT1* or *IKZF1* DNA methylation; no cycle threshold (Ct) value cut-offs were applied. Each PCR plate included three no-template control samples and a standard curve based on 0-2ng bisulphite converted fully methylated human DNA (Merck-Millipore, MA, United States) prepared in a background of nuclease-free water (Promega, WI, United States). The mass of methylated *BCAT1* and *IKZF1* DNA in each plasma specimen was determined from the batch specific standard curve. The level of methylation was expressed as the total mass of methylated (*BCAT1* plus *IKZF1*) DNA as a percentage of the total amount of recovered DNA per processed specimen.

### Statistical analyses

Subjects were recruited until at least 100 cancer cases had been identified (keeping 95 % CI of sensitivity estimates to less than 20 %) with at least 25 cases at each of stages I-III (to enable determination of the relationship between positivity rate and stage). The main outcome measure was positivity rate by diagnosis. GraphPad online scientific software tool, http://graphpad.com/scientific-software/, was used to calculate 95 % confidence intervals (binomial distribution assumed), Chi-square values (using 2x2 contingency tables without Yates’ correction) and McNemar’s test. Linear weighted Kappa statistic and odds ratios were calculated using www.vassarstats.net and www.medcalc.org/calc/odds_ratio.php, respectively.

Analysis of potential confounding co-variables was performed using a logistic generalised linear model fitted to a binary positivity variable (R package version 3.1.2) or by using a 2-sample z-test (two-tailed, 95 % significant level, http://www.socscistatistics.com/tests/ztest/Default2.aspx) on sample proportions (positive results observed in a given sample size). Continuous variables included age and DNA; dichotomous variables included smoking status, gender, and family CRC history.

An ANOVA Chi-square test (R version 3.1.2) was performed on assay positivity rates corrected for stage distribution in proximal and distal cancers using a generalised linear model with a logistic regression model fitted to two covariate models including stage and lesion, or lesion only.

The log values of the percentages of methylated *BCAT1* and *IKZF1* DNA measured in amount of DNA retrieved per processed specimens were used to create empirical density plots for three clinical classes: non cancer (all pathologies minus CRC cases), early stage cancer (Stage I + II) and late stage cancer (Stage III + IV). A minus infinity value was assigned to all cases with no methylation signal, whereas a Gaussian distribution was assumed for all non-zero values. By fitting Gaussian distribution curves to the empirical density plots, relative risk was calculated as the ratio of the conditional probability for early or late stage cancer compared to non-cancer based on the equation $\frac{\mathrm{P}\left(\left.\mathrm{X}=1\right|\left.\mathrm{Y}=1\right)\right.}{\mathrm{P}\left(\left.\mathrm{x}=0\right|\left.\mathrm{Y}=1\right)\right.}=\frac{{\mathrm{P}}_{11}}{{\mathrm{P}}_{01}}$, where X = 1 means cancer, X = 0 means no cancer and Y is the test result (positive (Y = 1) or negative (Y = 0)) at a given threshold value.

Reported p-values are 2-tailed and values <0.05 were considered statistically significant.

## Results

### Study subjects and cases

**Fig. 1:**
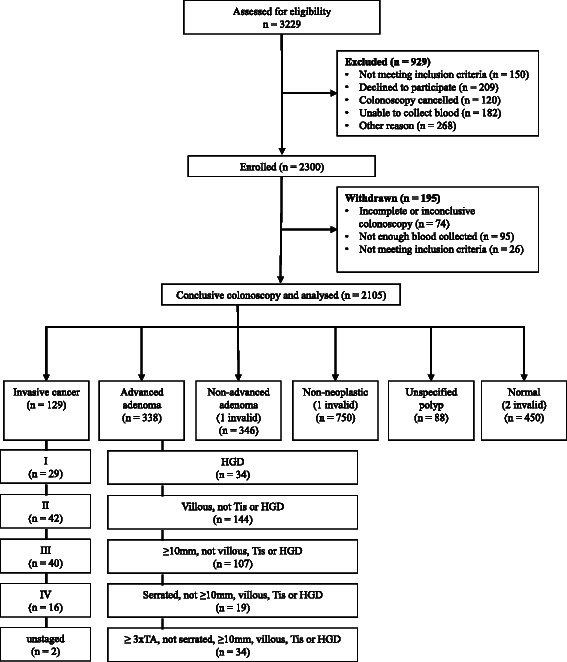
Disposition and outcomes of study volunteers approached for study inclusion. HGD: high-grade dysplasia, LGD: low-grade dysplasia, TA: tubular adenoma

Subjects were recruited from the Australian sites during the period September 2011 to May 2014 and from Dutch sites during July 2011 until September 2013 (see Additional file 2 for details). Figure 1 summarises the disposition of volunteers from initial approach through to diagnosis, including the reasons for exclusion or withdrawal. Sufficient plasma was collected prospectively as per protocol, i.e. following ingestion of bowel preparation but prior to colonoscopy, for almost all recruits (2078 of 2105, 99 %). Table 1 shows age and gender relative to principal diagnosis. Diagnoses in the 27 retrospective cases were 21 with cancer, 2 with diverticular disease, 1 with advanced adenoma, 1 with benign polyps and 2 with no evidence of pathologies.

**Table 1:** Demographic details for all eligible volunteers

Principal Diagnosis		Age (years)	Females	Males
	*N (%)*	$\tilde{x}{}^4\left( min\mathit{\hbox{-}} max\right)$	*N (%),* $\tilde{x}$ *age*	*N (%),* $\tilde{x}$ *age*
Total cases	2105	62 (33 - 90)	973 (46), 61	1132 (54), 63
No Neoplasia^1^	1291 (61)	61 (33 - 86)	673 (52), 60	618 (48), 61
Normal colon	452 (21)	58 (40 - 85)	259 (57), 57	193 (43), 60
Non-neoplastic pathology^2^	778 (37)	63 (40 - 86)	382 (49), 64	396 (51), 63
IBD	61 (3)	51 (33 - 86)	32 (53), 51	29 (48), 51
Adenoma	685 (33)	64 (40 - 85)	246 (36), 63	439 (64), 64
Non advanced	346 (17)	65 (40 - 85)	130 (38), 64	216 (62), 65
Advanced^3^	339 (16)	63 (41 - 85)	116 (34), 62	223 (66), 64
Cancer	129 (6)	69 (37 - 90)	54 (42), 68	75 (58), 69
Stage I	29 (1)	64 (45 - 86)	13 (45), 62	16 (55), 66
Stage II	42 (2)	72 (46 - 90)	17 (41), 75	25 (60), 72
Stage III	40 (2)	69 (39 - 88)	16 (40), 69	24 (60), 69
Stage IV	16 (1)	66 (37 - 88)	7 (44), 67	9 (56), 65
Unstaged	2 (0.1)	71 (57 - 85)	1 (50), 57	1 (50), 85

^1^All non-neoplastic cases, i.e. excluding only cases with adenomas or cancer. ^2^Including polyps (hyperplastic, unspecified, other polyps), angiodysplasia, haemorrhoids and diverticular disease. Excluding inflammatory bowel disease (IBD), which is shown separately. ^3^Includes two stage 0 (i.e. non-invasive) cancers. ${}^4\tilde{x}$; the median value

Cancer was the principal diagnosis in 6 % of all enrolled study subjects (129 of 2105 recruits) while adenoma (including stage 0 cancer) was diagnosed in 33 % of the recruits. Non-neoplastic pathologies (including IBD) were diagnosed in 40 % while 21 % recruits (452) showed no evidence of pathology in the colon or rectum. These phenotype frequencies reflect the recruitment strategy, which was designed to capture cases with a broad range of pathologies including all stages of neoplasia. More males (53.7 %) than females were recruited and more cancer patients were male (58.1 %) as would be expected [19].

### Assay performance estimates

The two-marker blood test was run successfully (i.e. meeting minimum quality control criteria) on 2127 samples, with 26 of these blood samples obtained after surgical resection of cancers. Table 2 shows the number of cases positive by one or both methylation markers according to diagnosis. Of the 129 cancer cases, 57 % were methylation positive for *BCAT1* and 48 % for *IKZF1*, with 66 % methylation positive by either gene. The true positive rate increased with stage for each marker and for the combined two-marker blood test (either methylation marker positive). Sensitivity estimates for the two-marker blood test for detection of earlier stage cancer (I or II) was 56 % (95 % CI: 44–68) and for later stage cancer (III + IV) was 79 % (95 % CI: 66–88), *p* = 0.009.

**Table 2:** Methylation marker performance by clinical findings, including selected sub-categories

Most advanced findings	No. (%)	Positivity Counts (%); 95 % CI				
		*BCAT1*	*IKZF1*	Either marker	OR (95 % CI)^1^	*X* ^2^
ALL CASES	2101	181 (9); 8-10	89 (4); 3-5	204 (10); 8-11		
Cancer	129 (6)	74 (57); 48 - 66	62 (48); 39 - 57	85 (66); 57 - 74	34 (20 - 59)**	241**
Stage I	29 (22)	7 (24); 10 - 44	8 (28); 13 - 47	11 (38); 21 - 58	11 (5 - 26)**	43**
Stage II	42 (33)	26 (62); 46 - 76	17 (40); 26 - 57	29 (69); 53 - 82	40 (18 - 86)**	16**
Stage III	40 (31)	27 (68); 51 - 81	22 (55); 38 - 71	29 (73); 56 - 85	47 (21 - 105)**	172**
Stage IV	16 (12)	13 (81); 54 - 96	15 (94);70 - 100	15 (94);70 - 100	266 (34-2101)**	158**
Unstaged	2 (2)	1 (50); 1 - 99	0 (0); 0 - 80	1 (50); 1 - 99	18 (1 - 293)**	8*
Early Stage (I + II)	71 (55)	33 (46); 35 - 59	25 (35); 24 - 47	40 (56); 44 - 68	23 (12 - 43)**	148**
Late Stage (III + IV)	56 (43)	40 (71); 58 - 83	37 (66); 52 - 78	44 (79); 66 - 88	65 (30 - 139)**	230**
Adv. adenoma^2^	338(16)	16 (5); 3 - 8	7 (2); 1 - 4	20 (6); 4 - 9	1.1 (0.6 - 2)	0.1
HGD	32 (9)	2 (6); 1 - 21	1 (3); 0.1 - 16	2 (6); 0.1 - 21	1.2 (0.3 - 5)	0.1
TVA^3^	144(43)	7 (5); 2 - 10	0 (0); 0 - 20	7 (5); 2 - 10	0.9 (0.4 - 2)	0.1
≥10mm^4^	107(32)	3 (3); 1 - 8	4 (4); 1 - 9	5 (5); 2 - 11	0.9 (0.3 - 2)	0.1
≥3 TAs (<10mm)	34 (10)	4 (12); 3 - 27	0 (0); 0 - 10	4 (12); 3 - 27	2.4 (1 - 7)	2.4
Serrated Adenoma	19 (6)	0 (0); 0 - 20	2 (11); 2 - 52	2 (11); 2 - 52	2 (0.5 - 10)	0.9
Non adv. adenoma	346(16)	23 (7); 4 - 10	2 (1); 0.1 - 2	23 (7); 4 - 10	1.3 (0.7 - 2)	0.6
No neoplasia	838(40)	46 (6); 4 - 7	15 (2); 1 - 3	52 (6); 5 - 8	1.2 (0.7 - 2)	0.4
IBD^5^	61 (3)	3 (5); 1 - 14	0 (0); 0 - 6	3 (5); 1 - 14	0.9 (0.3- 3)	0.01
Non neoplastic polyps^6^	296(14)	16 (5); 3 - 9	4 (2); 0.4 - 4	18 (6); 4 - 9	1.1 (0.6 - 2)	0.2
Hemorrhoids	288(60)	14 (5); 3 - 8	6 (2); 1 - 4	16 (6); 3 - 9	1.0 (0.5 - 2)	0.02
Angiodysplasia	11 (0.5)	2 (18); 2 - 52	0 (0); 0 – 28	2 (18); 2 - 52	4 (1 -19)	3
Diverticular disease	182(38)	11 (6); 3 - 11	5 (3); 1 - 6	13 (7); 4 - 12	1.3 (0.7 - 3)	0.8
Normal colon/rectum	450(21)	22 (5); 3 - 7	3 (1); 0 - 2	24 (5); 3 - 8	1	1

^1^Calculation of Odds Ratios (OR) or Chi-square (X^2^) values against normal colon/rectum; *P-values <0.05, **P-values <0.001; ^2^Advanced adenoma including Stage 0 cancers; ^3^Excluding HGD; ^4^no HGD or TVA; ^5^Inflammatory bowel disease ^6^Hyperplastic, unspecified and other polyps

*HGD* high-grade dysplasia, *TVA* tubulovillous adenoma, *TA* tubular adenoma, *IBD* inflammatory bowel disease

By contrast, sensitivity estimates for adenomas of any type were low, at 6 % (95 % CI: 4–9) for advanced adenoma and 7 % (95 % CI: 4–10) for non-advanced adenoma. These estimates were not significantly different compared to positivity rates in those with a normal colon or benign pathology (Table 2, *p* > 0.05).

Specificity estimates for the combined two-marker blood test were 94 % (95 % CI: 93–95, 1288 non-neoplastic cases) to 95 % (95 % CI: 92–97, 450 cases with no evidence of disease).

### Concordance between methylation markers

Methylated *IKZF1* DNA was typically detected at a lower rate in blood compared to methylated *BCAT1* DNA across all diagnostic sub-classes. Concordance between the two markers is shown for selected clinical phenotypes in Table 3. For those with cancer, 51/129 (40 %) were concordant and 34/129 discordant (26 %), with *BCAT1* detecting most of the discordant cases (23/34, 68 %) (McNemar’s, *p* = 0.06). The linear weighted Kappa statistic as a measure of agreement was 0.476 for cancer cases (95 % CI: 0.327–0.625).

**Table 3:** Methylation marker concordances for selected phenotypes

Most advanced findings	No.	No. *BCAT1/IKZF1* positive				*P*-value^1^
		+/+	+/-	*-/+*	*-/-*	
Cancer	129	51	23	11	44	0.059
Advanced adenoma	338	3	13	4	318	0.052
Non-neoplastic pathologies	838	9	37	6	786	<0.0001
Normal colon/rectum	450	1	21	2	426	0.0002

^1^McNemar t-test

In subjects with no evidence of pathologies in colon and rectum, only one case of the 24 positive results showed concordance between the methylation markers with *BCAT1* being responsible for most (21/23) of the discordant cases (McNemar’s, *p* = 0.0002). Linear weighted Kappa measure of agreement was 0.07 (95 % CI: 0–0.213).

### Other factors related to marker positivity

The influence of recruitment site, age, gender, smoking status, family history of CRC and amount of cell free DNA on assay positivity was assessed. Recruitment site (see Additional file 2), gender, family history of CRC (see Additional file 3) and age (see Additional file 4) were not significant predictors of assay positivity (*p* > 0.05).

For 286 cases with known smoking habits, 62 % were current smokers. Excluding the 16 CRC cases that smoked, 11/165 smokers were methylation positive compared to 11/105 non-smokers (Fisher’s p-value = 0.362).

The majority of processed specimens had cell free DNA amounts of 1.6-2.5ng per mL plasma (95 % CI). There was no significant difference in levels of cell-free DNA between all subjects without CRC and cancer cases of stages I to III, however some stage IV cancer cases had a significantly higher amount of DNA (see Additional file 5, *p* > 0.0001). Excluding cases with cancer, the average amount of cell-free DNA was 2.1ng/mL (95 % CI: 1.9-2.2). Higher DNA amounts (>3ng/mL) were observed in 192 of 1972 non-CRC cases (9.7 %), of which 19 (10 %) were two-marker blood test positive. Increased DNA amounts was associated with an increased chance of a positive result, as the odds ratio for positivity increased 2.7-fold for each increment of one in log (DNA pg/mL), *p* value <0.0001.

### Distal versus proximal disease

The estimated positivity rates for proximal (60 %) and distal (67 %) cancers were not significantly different (Chi-square test, *p* value = 0.603). Cancer location, corrected for stage distribution, did not influence detection of markers in blood (Additional file 3, *p* value = 0.555).

### Tumour invasiveness and detectability

**Fig. 2:**
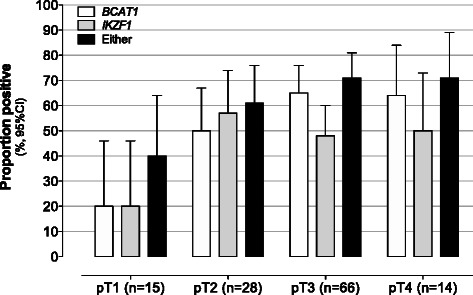
Marker positivity rates versus cancer invasiveness. The proportion (%) of cancer cases (pT staging) positive for *BCAT1* (white bars), *IKZF1* (grey bars) or either marker (black bars)

The relationship between detection of methylated *BCAT1* and *IKZF1* DNA in blood and degree of invasiveness (by pT stage) for cancers is shown in Fig. 2. Although not statistically significant (ANOVA with Tukey post-hoc test), a positive trend was observed between positivity rate and pT stage (degree of invasion) for each marker, and the two-marker blood test.

### Quantitative testing and cancer stage prediction

**Fig. 3:**
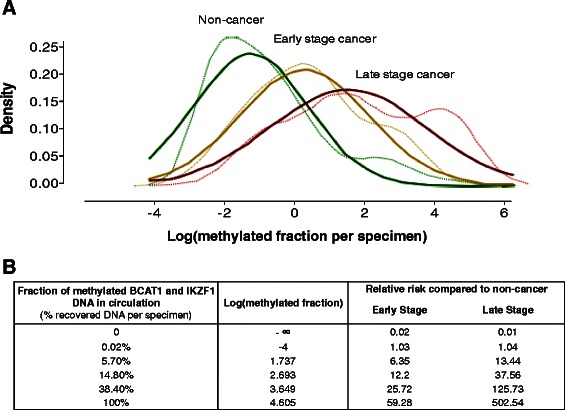
Relative risk prediction based on quantitative assessment of methylation. **a** The amount of methylated *BCAT1* and *IKZF1* as a percentage of total DNA per specimen was used to compute empirical density plots (thin lines) and fitted Gaussian curves (bold lines) from non-cancers (green), early stage cancer (yellow, stage I + II) and late stage cancer (red, stage III + IV). **b** Relative risk calculations for a given value of methylated *BCAT1* and *IKZF1* DNA. The minus infinity (-∞) is the log of no methylation (zero values)

As per study protocol, the two-marker blood test performance estimates have been qualitatively reported as any detectable signal for methylated *BCAT1* and/or *IKZF1* DNA. However, positive qPCR methylation results can also be reported quantitatively as the fraction of methylated *BCAT1* plus *IKZF1* DNA measured in the total yield of DNA isolated per specimen. We modelled the relationship between disease severity (non-cancer, early stage cancer and late stage cancer) and the fraction of methylated *BCAT1* and *IKZF1* DNA. Figure 3a shows that the fraction of methylated *BCAT1* and *IKZF1* DNA in blood increased as a function of degree of invasiveness. The generated models were used to calculate the relative risk of disease (early stage or late stage cancer) compared to non-cancer for a given methylation fraction value. The models indicated a low relative risk of having cancer if no methylation was detected. For a specimen containing approximately 5 % methylated *BCAT1* and *IKZF1* DNA the models estimate a relative risk of 5 for having early stage cancer (Fig. 3b). On the other hand, the relative risk of being late stage cancer given a specimen with approximately 40 % methylation is 125.

### Marker methylation levels after resection

Of the 129 cancer cases, a post-resection sample was available for 12 of the 85 cases with a positive two-marker blood result at initial diagnosis, and for 14 of the 44 cases with a negative result at diagnosis. As can be seen in Table 4, ten of twelve initially positive cases became negative after resection. We note that the *BCAT1* and *IKZF1* methylation levels <5 % are values obtained from extrapolation due to these methylation signals being below the linear range of the qPCR assay. Of the 14 cases that were negative at diagnosis, all but one remained negative after resection (data not shown).

**Table 4:** Blood methylation levels in 12 CRC cases positive before and after tumour resection

Case characteristics		Proportion of methylated *BCAT1* and *IKZF1*^*1*^ (% of total yield)		
Tumour location	Stage	Before resection	After resection	Δ Days
Sigmoid	l	<0.00001	0	140
Splenic flexure	l	<0.00001	0	48
Caecum	llA	1.8	0	115
Ascending	llA	0.7	<0.00001	70
Sigmoid	llA	5.6	0	162
Sigmoid	llA	2.6	0	64
Ascending	llA	1.7	0	83
Ascending	llA	1.4	0	39
Caecum	lllA	5.0	0	58
Rectum	lllA	0.8	0	47
Ascending	lllB	0.9	0	50
Sigmoid	lllC	0.6	0.6	157

^1^The lower limit of the linear range for the qPCR assay was 100pg per reaction. The average DNA amount per reaction was 2 ng, thus methylation levels estimated to be <5% but above zero are extrapolated and most likely inaccurate

## Discussion

By estimating the true- and false-positive rates of the two-marker blood test for screen-relevant stages of colorectal neoplasia, we have been able to determine that a blood test detecting methylated *BCAT1* and *IKZF1* DNA facilitates identification of cases with CRC relative to other clinical states encountered in the colon and rectum.

We estimated an overall sensitivity for CRC of 66 % (*n* = 129, 95 % CI: 57–74), with better detection of later versus earlier stage cancers (79 % compared to 56 %). This overall sensitivity is within the upper half of the reported sensitivity range of 37–79 % for guaiac FOBT (gFOBT) in populations such as we have studied here or in true screening populations [20]. Despite low sensitivity in the original gFOBTs, RCTs still showed effectiveness of the technology in reducing mortality from CRC [3, 4]. In a micro-simulation model to estimate gFOBT sensitivity for CRC from the first three RCTs it was estimated that gFOBT sensitivity was 51 % for the stages of clinical diagnosis and 19 % for early stage cancer [21]. This implies an adequate sensitivity of the two-marker blood test for reducing CRC mortality if used as a screening test but this prediction requires validation in true screening populations. The two-marker blood test has a low sensitivity for advanced adenomas and should not be expected to impact on CRC incidence as seen with certain faecal immunochemical tests (FIT) which have sensitivity for advanced adenomas in the range 29–45 % [22, 23].

Impact of a screening test on population mortality from CRC is not dependent only on test accuracy but also on participation rates. Given the stated preference of a typical screening population for the idea of a blood test over a faecal test [8], including a subset who had already undertaken screening with FIT [9], one could predict that even if a lesser sensitivity were to be confirmed for the two-marker blood test when validated in true screening populations, a participatory advantage might counterbalance this.

The earlier estimates of sensitivity for cancer and advanced adenoma for methylated Septin 9 (*SEPT9*) were comparable to those seen with our two-marker blood test [12, 24–26], although a large-scale study in a screening population returned a cancer sensitivity of 51 % [13]. The reported observed sensitivity for stage I cancer of 36 % was almost identical to ours (38 %), while neither study achieved a sensitivity of 10 % for advanced adenomas. Whether there is complementarity of our markers with *SEPT9* for cancer detection is unclear at present and warrants study.

To determine whether this apparent lower sensitivity for early stage cancer and adenomas was a function of the assay or a biologically-determined issue, we examined the relationship of positivity to tumour depth of invasion and modelled the biomarker mass relative to risk for different stages of neoplasia. A trend was observed between assay positivity and degree of cancer invasiveness (pT stage), which was not affected by the colonic location or other potential variables examined. By modelling the stage of neoplasia relative to marker mass, we show the potential for using the measured percentage of methylated *BCAT1* and *IKZF1* DNA in blood to estimate the relative risk of disease severity. Given that the assay is sensitive at the limits of detection to 6 DNA copies per mL of plasma (Additional file 1), some stage I cancers might escape detection due to very low amount of tumour-derived DNA reaching the blood [27, 28]. As adenomas are non-invasive, this might account for a biological limitation in the capacity of blood tests to detect adenomas.

If methylated DNA biomarkers are fundamentally disadvantaged compared to FIT in detection of advanced adenomas, then what is their place in CRC screening? Where programs seek to detect just a proportion of cancers with high efficiency and low colonoscopy rates [29], a blood DNA test might be acceptable as a frontline screening test if a participatory advantage can be demonstrated in practice. It seems more likely that at the present moment, blood DNA tests will be applicable to people where an FOBT is inappropriate due to bleeding benign lesions or as a second line rescue strategy for engaging those in screening who otherwise reject the faecal test.

The false-positive rate for the two-marker blood test provides insight into specificity and the factors that might influence it, and hence cost. Our observed specificity was 94–95 %, which was slightly better than the reported 91 % for *SEPT9* [13]. Smoking, family history of CRC, gender and age were not significant predictors of assay positivity. There was no significant difference in DNA yields between non-CRC and cases with stage I-III cancers, however higher yields were observed for some stage IV cancers as reported previously [12]. Further, we did observe an increase in assay positivity in non-neoplastic cases where recovered DNA exceeded 3ng/mL. Given the results of the technical assessment (Additional file 1), it seems likely that the false-positives (as determined by colonoscopy) reflect a true appearance of methylated *BCAT1* and *IKZF1* DNA. Longitudinal follow-up studies are required to understand whether the low false-positive rate in healthy cases reflects chance events (i.e. methylation of *BCAT1* DNA especially) of no consequence, or an early indication of colorectal neoplasia and/or other extra-colonic cancers.

The biological functions of *BCAT1* and *IKZF1* are not well understood, but both genes are involved in tumour growth and invasiveness [30, 31]. Both genes have been demonstrated to be hypermethylated in several cancers including CRC [10, 32]. Emerging data imply that *IKZF1* is a crucial player in proper regulation of proliferation and differentiation by controlling the activity of a small set of genes including notch [33–36] which plays a crucial role in the self-renewing process of colon crypt stem cells [37, 38].

The disappearance of circulating methylated *BCAT1* and *IKZF1* DNA after tumour resection in 10 of 12 cancer cases shows that detection of methylated *BCAT1* and *IKZF1* DNA in the blood reflects the presence of CRC rather than a risk of developing CRC. The half-life of free DNA in the blood is reportedly short at ~2 hours [39], but 2 CRC cases remained positive for methylation even 5 months after resection. Longer follow-up is needed in the two cases with persisting methylation signal to understand the reason, as it is possible they were not cured of their cancer. Similar to observations made for other CRC methylation markers, these data suggest that the two-marker blood test may be useful to monitor tumour recurrence and adequacy of resection and/or initial therapy [40].

There are several additional limitations with this study. The estimated sensitivities and specificities might not apply to screen-detected lesions, and comparison to other non-invasive screening tests has yet to be undertaken in this context. Actual test positivity rates in a true screening population cannot be reliably estimated from this study and so the consequences for colonoscopy follow-up rates are uncertain. As with all other DNA tests under consideration for CRC screening, how specific they are for colorectal as opposed to other organ cancers remains uncertain and long-term follow-up of false-positive cases is required.

## Conclusion

Accuracy of the two-marker blood test approximates that of the less-sensitive gFOBT [19]. Consequently it is now justifiable to proceed to prospective evaluation in a true screening population relative to FIT. At present, the likely use of this two-marker blood test for screening seems most appropriate in a rescue strategy for those refusing more sensitive RCT-proven methods such as FIT, flexible sigmoidoscopy or colonoscopy.

## Additional files

Additional file 1: **Detailed assay protocol.** **Table S1.** DNA sequences for the oligonucleotides used in the 2-marker blood test qPCR assay. **Table S2.** qPCR cycling conditions. (PDF 148 kb)
Additional file 2: **Recruitment details for participating clinical sites.** **Table S3.** Distribution of recruits from the four hospitals participating in the study. (PDF 97 kb)
Additional file 3: **Co-variable analysis.** **Table S4.** Gender. **Table S5.** Family CRC history. **Table S6.** Assay positivity rates relative to tumour location. The proportion of positivity assay results was modelled (R package version 3.1.2) using a generalised linear model (glm) with a logit link (logistic regression model) fitted to two covariate models including stage and lesion or stage only. An ANOVA with a Chi-square test demonstrated that the two models were not statistically different (*p* value = 0.555). (PDF 88 kb)
Additional file 4: **Age versus assay positivity.** **Figure S1.** The proportion of positive blood results were calculated for <50, 50-54, 55-59, 60-64, 65-69, 70-74, 75-80 and >80 years of age. The binomial standard deviation was calculated using the formula $\mathrm{S}\mathrm{E}\mathrm{p}=\sqrt{\mathrm{p}\left(1\hbox{-} \mathrm{p}\right)/\mathrm{n}}$, where *p* = proportion of positive results, *n* = sample size (https://www.easycalculation.com/statistics/standard-error-sample-proportion.php). A two-sample Z-test two-tailed, 95 % significant level was performed on the terminal groups less than 50yrs of age versus more than 80yrs of age (the age span in study cohort) and 50-54yrs vs 75-80yrs (screen-eligible age) based on the assumption that if there was an age trend then that would be most pronounced in ‘young’ versus ‘old’. (A) non-neoplastic controls (*n* = 1288); (B) cancer (*n* = 129). (TIFF 2521 kb)
Additional file 5: **Circulating cell-free DNA levels versus assay positivity.** **Figure S2.** Cumulative plots for DNA amount (log2, ng/mL), for (A) non-cancer and Stage I-III and (B) Stages I to III as well as the individual cancer stages (I to IV). There was no significant difference in DNA amounts between non-cancer and cancer stages I to III (Kolmogorov-Smirnov test, max deviation: 0.035, *p* = 0.1785), whereas a number of stage IV samples had high DNA yields (max deviation = 0.513, *p* value = 0.0001). (TIFF 2412 kb)

## Abbreviations

CRC: Colorectal cancer
*BCAT1*: Branched chain amino-acid transaminase 1
*IKZF1*: IKAROS family zinc finger 1
qPCR: quantitative PCR
PCR: Polymerase chain reaction
HGD: High-grade dysplasia
LGD: Low-grade dysplasia
TVA: Tubulovillous adenoma
TA: Tubular adenoma
IBD: Inflammatory bowel disease
RCT: Randomised controlled trial
FOBT: Faecal occult blood test
FIT: Faecal immunochemical test
Ct: Cycle threshold
