# Supplementary material for: Evaluation of an assay for methylated BCAT1 and IKZF1 in plasma for detection of colorectal neoplasia
Source: BMC Cancer. 2015 Oct 6;15:654. doi: 10.1186/s12885-015-1674-2 (PMC4596413; doi:10.1186/s12885-015-1674-2)
Supplement: Additional file 1: — Detailed assay protocol. Table S1. DNA sequences for the oligonucleotides used in the 2-marker blood test qPCR assay. Table S2. qPCR cycling conditions. (PDF 148 kb) [file 12885_2015_1674_MOESM1_ESM.pdf]

Additional File 1

Detailed assay protocol

DNA Extraction: Plasma samples less than 4.3mL (but >3.9ml) were supplemented with sterile 1X PBS where necessary. Circulating cell-free DNA was extracted using the QIASymphony CNA kit (cat. No.:1072879, Qiagen) on the QIASymphony SP instrument installed with the CF4000 software and Ab-0785 labware. Elution volume was set to 85μL. Samples were analysed in batches including 22 clinical samples and two process controls. The methylation positive/ACTB positive control consisted of pooled human donor plasma (Bioreclamation, NY, USA) spiked with 1250pg/mL of sonicated, enzymatically-methylated human genomic DNA (Millipore, MA, USA). The methylation negative/ACTB-positive controls were pooled human donor plasma. The process controls were made as 2 litre batches and stored as 4.5mL aliquots at -80°C until further use.

Bisulphite conversion & purification: The extracted DNA was bisulphite-converted using the EpiTECT Fast Bisulfite Conversion kit (Qiagen) as per manufacturer's instruction and subsequently purified using the same kit on a QIACube instrument as per manufacturer's recommendation (Qiagen) except for a few modifications: The spin columns from the QIASymphony CNA kit were used in place of the spin columns provided in the EpiTECT Plus Bisulphite kit and the EpiTECT Plus Binding buffer was prepared with isopropanol instead of ethanol. Elution volume was set to 40 μL and the eluate was manually re-applied to the spin columns and re-centrifuged, to improve DNA yield (data not shown).

Real-time PCR analysis: The methylation specific real-time PCR assay comprised oligonucleotides for simultaneous amplification of CpG methylated regions spanning 102- or 95-nucleotides either within, or just upstream of, the first exon of the *BCAT1* and *IKZF1* genes, respectively. Oligonucleotides were manufactured and purified by reverse-phase HPLC (Integrated DNA Technologies, Iowa, United States). Oligonucleotide sequences for the three PCR targets *ACTB* (control), *BCAT1* and *IKZF1* are provided in Table S1. Aliquots of 10X Oligo

mixture was made and stored at -20°C until further use. Each processed plasma sample was analysed as three PCR replicates by analysing 12μL of bisulphite converted DNA in a total PCR volume of 30μL including 15μL of 2X QuantiTECT NoROX mastermix (Qiagen) and 3μL 10X Oligo Mix (resulting in final concentrations of 200nM and 100nM for primers and hydrolysis probes, respectively. Each PCR run included three no-template control samples. The real-time PCR assay was performed on a Light Cycler 480 II instrument (Roche Diagnostics, IN, USA), Table S2. Colour compensation was performed and applied to each run as per the manufacturer's instructions. Cycle threshold (Ct) values were calculated using the absolute quantification - second derivative maximum algorithm provided with the LC480 software.

Further technical information for the 2-marker qPCR assay

A technical assessment of the 2-marker qPCR assay indicated no false-positive signal for up to ~30,000 copies (~92ng) of unmethylated BCAT1 and IKZF1 DNA targets (synthetically engineered, data not shown). The limit of detection (LOD) was determined by spiking 4mL healthy donor plasma specimens with fully-methylated DNA (Merck-Millipore) at 1.6-, 3.1, 6.3, 12.5, 25 and 50 pg/mL and estimated to be 18pg/mL using a 95% probability criteria (data not shown). The estimated LOD is equivalent to ~6 copies (3 diploid cell genomes) of DNA per mL of plasma.

Table S1. Oligonucleotide sequences

FWD	5'- GTTTTTTTGTTGATGTAATTCGTTAGGTC (*BCAT1*)<br>5'- GACGACGTATTTTTTTCGTGTTTC (*IKZF1)*<br>5'- GTGATGGAGGAGGTTTAGTAAGTT (*ACTB*)
REV	5'- CAATACCCGAAACGACGACG (*BCAT1*)<br>5'- GCGCACCTCTCGACCG (*IKZF1*)<br>5'-AATTACAAAAACCACAACCTAATAAA (*ACTB*)
Probes	HEX-TTCGTCGCGAGAGGGTCGGTT- 3IAbFQSp (*BCAT1*)<br>FAM- TTTGTATCGGAGTAGCGATTCGGGAG- 3IAbFQSp (*IKZF1*)<br>Texas Red- ACCACCACCCAACACACAATAACAAACACA-3IAbRQSp (*ACTB*)

FWD: forward primers, REV: reverse primers, Probes: 5'-hydrolysis probes

Table S2. PCR Cycling Conditions

Cycles	Analysis	Temp	Acquisition	Hold[2]	Ramp Rate
1	None	95°C	None	0:15:00	4.4°C/s
50	Quantification[1]	95°C	None	0:00:15	4.4°C/s
		62°C	Single	0:00:40	2.2°C/s
Cooling:	None	40°C	None	0:00:10	2.2°C/s

[1]Detection channels: *IKZF1*, FAM (465-510nM); *BCAT1* HEX (533-580nM); *ACTB*, Texas Red (533-610nM), [2]Hr:mm:ss
