# Supplementary material for: Evaluation of an assay for methylated BCAT1 and IKZF1 in plasma for detection of colorectal neoplasia
Source: BMC Cancer. 2015 Oct 6;15:654. doi: 10.1186/s12885-015-1674-2 (PMC4596413; doi:10.1186/s12885-015-1674-2)
Supplement: Additional file 2: — Recruitment details for participating clinical sites. Table S3. Distribution of recruits from the four hospitals participating in the study. (PDF 97 kb) [file 12885_2015_1674_MOESM2_ESM.pdf]

**Table S3 Recruitment details for the participating hospitals.**

| | Recruitment Centres | | | | |
|---|---|---|---|---|---|
| | FMC[1] | RGH[2] | AMC[3] | Flevo[4] | Total |
| **Recruits** (*No.* (Age[5])) | 1422 (62) | 347 (64) | 162 (63) | 170 (63) | 2101 |
| **Females** (No. (%), Age[5]) | 656 (46), 61 | 170 (49), 65 | 73 (45), 62 | 72 (42), 64 | 971 |
| **Males** (No. (%), Age[5]) | 766 (54), 62 | 177 (51), 63 | 89 (55), 64 | 98 (58), 62 | 1130 |
| **Primary Diagnosis** | | | | | **Chi square[6]** |
| **Cancer** (No., (%)) | 58 (4) | 12 (3.5) | 22 (14) | 37 (22) | 1.4142, n.s |
| # Positive blood results (%), 95%CI | 35 (60), 47-73 | 7 (58), 28-85 | 12 (55), 32-76 | 31 (84), 68-94 | |
| **Adenoma** (No., (%)) | 421 (30) | 106 (31) | 76 (47) | 81 (48) | 1.754, n.s |
| # Positive blood results (%), 95%CI | 29 (7), 5-10 | 8 (8), 3-14 | 2 (3), 0.3-9 | 4 (5), 1-12 | |
| **Non-neoplastic** (No., (%)) | 631 (44) | 181 (52) | 15 (9) | 11 (6) | 2.597, n.s |
| # Positive blood results (%), 95%CI | 36 (6), 4-8 | 13 (7), 4-12 | 0 (n/a), 0-22 | 3 (27), 6-61 | |
| **No evidence of disease** (No., (%)) | 312 (22) | 48 (14) | 49 (27) | 41 (24) | 7.008, n.s |
| # Positive blood results (%), 95%CI | 9 (3), 1-5 | 2 (4), 0.5-14 | 9 (18), 9-32 | 4 (10), 3-23 | |

[1] Flinders Medical Centre [2]Repatiation General Hospital, [3]Academic Medical Centre, [4]Flevo Hospital, [5] Median age in years. [6]Based on 4x2 contingency tables using the overall positive rate measured in the study population to calculate the expected positive rates across each collection site. A Chi Square statistic was performed on observed versus expected positivity rates using a 0.05 significance value. n.s = not statistically significant.
