# Supplementary material for: Evaluation of an assay for methylated BCAT1 and IKZF1 in plasma for detection of colorectal neoplasia
Source: BMC Cancer. 2015 Oct 6;15:654. doi: 10.1186/s12885-015-1674-2 (PMC4596413; doi:10.1186/s12885-015-1674-2)
Supplement: Additional file 3: — Co-variable analysis. Table S4. Gender. Table S5. Family CRC history. Table S6. Assay positivity rates relative to tumour location. The proportion of positivity assay results was modelled (R package version 3.1.2) using a generalised linear model (glm) with a logit link (logistic regression model) fitted to two covariate models including stage and lesion or stage only. An ANOVA with a Chi-square test demonstrated that the two models were not statistically different (p value = 0.555). (PDF 88 kb) [file 12885_2015_1674_MOESM3_ESM.pdf]

Table S4. Gender versus assay positivity

	Total	Female	Male	Z-score[1]
		No. +ve/Tot	No. +ve/Tot	
**Non-cancer**	1972	49/918	70/1054	-1.213, p = 0.226
**Cancer**	129	36/54	49/75	0.1576, p = 0.873

[1] http://www.socscistatistics.com/tests/ztest/Default2.aspx

Table S5. Family CRC history and assay positivity

2039 records of family history	No.	Blood test positivity<br>No. (%); 95%CI	Z-score[1]
**Cancer cases**	87	51 (59); 48-69	
No family CRC history	82	48 (59); 47-69	-0.065, p = 0.952
Family CRC history	5	3 (60); 15-95	
**Non-Cancer cases**	1952	118 (6); 5-7	
No family CRC history	1629	103 (6); 5-8	1.1566, p = 0.246
Family CRC history	323	15 (5); 3-8	

[1] http://www.socscistatistics.com/tests/ztest/Default2.aspx

Table S6. Assay positivity relative to tumour location – corrected for stage

Location	Stage I	Stage II	Stage III	Stage IV	Unstaged
Distal	16	16	21	10	1
Proximal	13	21	14	3	0
n/a	0	5	5	3	1
