# Supplementary figures and images for: Evaluation of an assay for methylated BCAT1 and IKZF1 in plasma for detection of colorectal neoplasia
Source: BMC Cancer. 2015 Oct 6;15:654. doi: 10.1186/s12885-015-1674-2 (PMC4596413; doi:10.1186/s12885-015-1674-2)

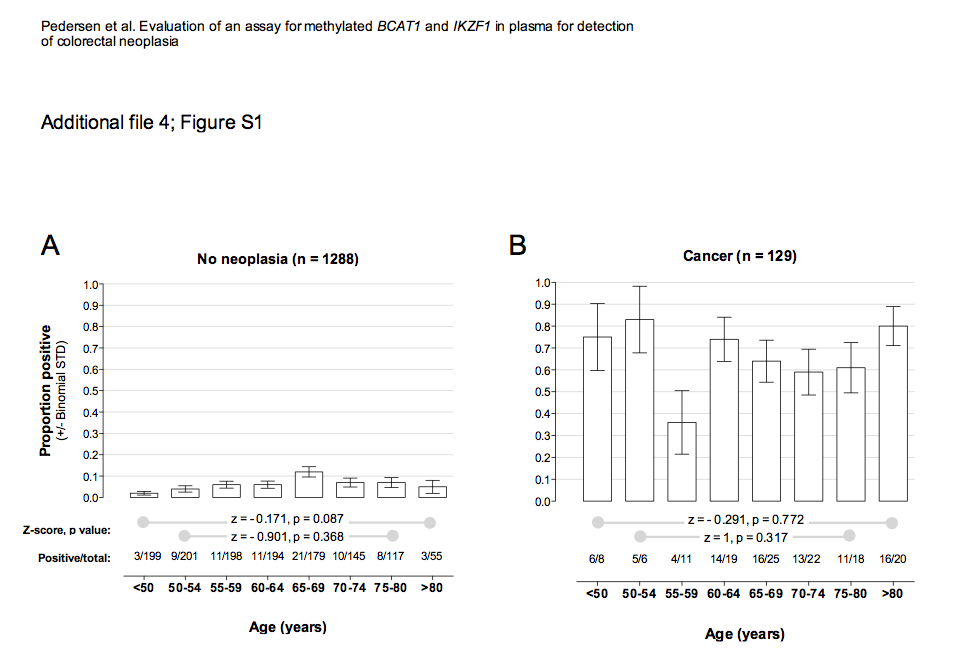

Supplement: Additional file 4: — Age versus assay positivity. Figure S1. The proportion of positive blood results were calculated for <50, 50-54, 55-59, 60-64, 65-69, 70-74, 75-80 and >80 years of age. The binomial standard deviation was calculated using the formula $\mathrm{S}\mathrm{E}\mathrm{p}=\sqrt{\mathrm{p}\left(1\hbox{-} \mathrm{p}\right)/\mathrm{n}}$SEp=p1‐p/n, where p = proportion of positive results, n = sample size (https://www.easycalculation.com/statistics/standard-error-sample-proportion.php). A two-sample Z-test two-tailed, 95 % significant level was performed on the terminal groups less than 50yrs of age versus more than 80yrs of age (the age span in study cohort) and 50-54yrs vs 75-80yrs (screen-eligible age) based on the assumption that if there was an age trend then that would be most pronounced in ‘young’ versus ‘old’. (A) non-neoplastic controls (n = 1288); (B) cancer (n = 129). (TIFF 2521 kb) [file 12885_2015_1674_MOESM4_ESM.tiff]

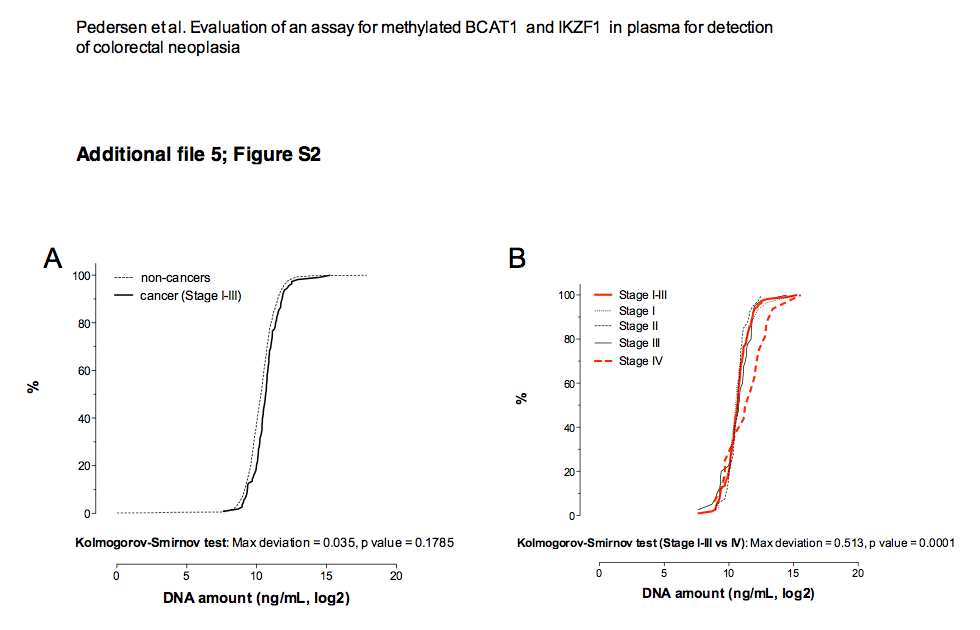

Supplement: Additional file 5: — Circulating cell-free DNA levels versus assay positivity. Figure S2. Cumulative plots for DNA amount (log2, ng/mL), for (A) non-cancer and Stage I-III and (B) Stages I to III as well as the individual cancer stages (I to IV). There was no significant difference in DNA amounts between non-cancer and cancer stages I to III (Kolmogorov-Smirnov test, max deviation: 0.035, p = 0.1785), whereas a number of stage IV samples had high DNA yields (max deviation = 0.513, p value = 0.0001). (TIFF 2412 kb) [file 12885_2015_1674_MOESM5_ESM.tiff]
